# A Method for the Simultaneous Estimation of Selection Intensities in Overlapping Genes

**DOI:** 10.1371/journal.pone.0003996

**Published:** 2008-12-22

**Authors:** Niv Sabath, Giddy Landan, Dan Graur

**Affiliations:** Department of Biology and Biochemistry, University of Houston, Houston, Texas, United States of America; University of Oxford, United Kingdom

## Abstract

Inferring the intensity of positive selection in protein-coding genes is important since it is used to shed light on the process of adaptation. Recently, it has been reported that overlapping genes, which are ubiquitous in all domains of life, seem to exhibit inordinate degrees of positive selection. Here, we present a new method for the simultaneous estimation of selection intensities in overlapping genes. We show that the appearance of positive selection is caused by assuming that selection operates independently on each gene in an overlapping pair, thereby ignoring the unique evolutionary constraints on overlapping coding regions. Our method uses an exact evolutionary model, thereby voiding the need for approximation or intensive computation. We test the method by simulating the evolution of overlapping genes of different types as well as under diverse evolutionary scenarios. Our results indicate that the independent estimation approach leads to the false appearance of positive selection even though the gene is in reality subject to negative selection. Finally, we use our method to estimate selection in two influenza A genes for which positive selection was previously inferred. We find no evidence for positive selection in both cases.

## Introduction

Overlapping genes were first discovered in viruses [1] and later in all cellular domains of life [2]–[4]. The percentage of overlapping genes in a genome varies across species: 5–14% in vertebrates [5], 10–50% in bacteria [6], and up to 100% in viruses (e.g., hepatitis B virus)[7]. Overlapping genes were suggested to have multiple functions such as regulation of gene expression [8], translational coupling [9], and genome imprinting [10]. In addition, overlapping genes were hypothesized to be a means of genome size reduction [11], as well as a mechanism for creating new genes [12].

The interdependence between two overlapping coding regions results in unique evolutionary constraints [13], [14], which vary among overlap types [13]. Several attempts at estimating selection intensity in overlapping genes have been made [15]–[26]. In some studies, one gene was found to exhibit positive selection while the overlapping gene showed signs of strong purifying selection (e.g., [15]). Inferences of positive selection in overlapping genes have been questioned [19], [21], [24], mostly because ignoring overlap constraints might bias selection estimates. Rogozin et al. [27] tried to overcome this problem by focusing on sites in which all changes are synonymous in one gene and nonsynonymous in the overlapping gene.

A model for the nucleotide substitutions in overlapping genes was introduced by Hein and Stovlbaek [28], who followed approximate models for non-overlapping genes that classify sites according to degeneracy classes [29]–[31]. This model was later incorporated into a method for annotation of viral genomes [32]–[34], and recently used for estimating selection on overlapping genes [35]. The main weakness of approximate methods is that it assumes a constant degeneracy class for each site, whereas degeneracy changes over time as substitutions occur. Pedersen and Jensen [36] suggested a non-stationary substitution model for overlapping reading frames that extended the codon-based model of Goldman and Yang [37]. This model encompasses the evolutionary process more accurately than the approximate model [28] by accounting for position dependency of each site in an overlap region [36]. However, this improvement disallowed the straightforward estimation of parameters and forced the authors to apply a computationally-expensive simulation procedure [36]. Surprisingly, these models for nucleotide substitutions in overlapping genes were rarely cited, not to mention used, by the majority of studies estimating selection in overlapping genes. One reason that these methods were seldom used might be the lack of an accessible implementation.

Here, we describe a non-stationary method, similar to that of Pedersen and Jensen [36]. Our method simplifies selection estimation and avoids the need for costly simulation procedure. We test our method by simulating the evolution of overlapping genes of different types and under various selective regimes. Further, we describe the nature and magnitude of the error when selection is estimated as if the genes evolve independently. Finally, we use our method to estimate selection in two cases for which independent estimation has previously yielded indications of positive selection.

## Methods

**Figure 1:**
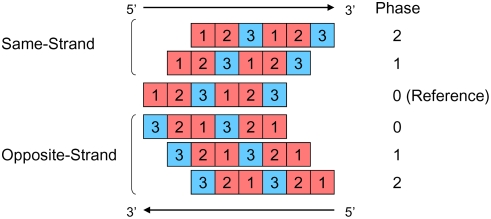
Orientations and phases of gene overlap. Genes can overlap on the same strand or on the opposite strand. The reference gene in a pair of overlapping genes is called phase 0. Same-strand overlaps can be in two phases (1 and 2); opposite-strand overlaps can be in three phases (0, 1, and 2). First and second codon positions, in which ∼5% and 0% of the changes are synonymous, are marked in red. Third codon positions, in which ∼70% of the changes are synonymous, are marked in blue.

A gene can overlap another on the same strand or on the opposite strand. Each overlap orientation has 2 or 3 possible overlap phases (Figure 1). To understand the consequences of estimating selection pressures on overlapping genes as if they are independent genes, let us consider a simplified view of the genetic code, in which all changes in first and second codon positions are nonsynonymous and all changes in third codon position are synonymous. (In reality, the proportions of changes that are synonymous are ∼5%, 0%, and ∼70% for the first, second, and third codon positions, respectively). From Figure 1 we see that in all overlap types, but one (opposite-strand phase 2), all synonymous changes in one gene are nonsynonymous in the overlapping gene, while half of the nonsynonymous changes are synonymous in the overlapping gene. Since the rate of synonymous substitutions is usually higher than that of nonsynonymous substitutions, ignoring overlap constraints would result in the underestimation of the rate of synonymous substitutions. (In the case of opposite-strand phase-2 overlaps, ignoring the overlap would result in the underestimation of nonsynonymous substitutions rate.) The bias in the estimation would be correlated with the strength of purifying selection on the overlapping gene. Thus, a false inference of positive selection is likely for genes under relaxed purifying selection when the overlapping gene is under strong purifying selection.

### Goldman and Yang's [37], [38] method for the estimation of selection intensity in non-overlapping coding sequences

The most commonly used method for estimating selection intensity on protein coding genes fits a Markov model of codon substitution to data of two homologous sequences [37], [38]. The codon-based model of nucleotide substitution is specified by the substitution-rate matrix, *Q_codon_* = {*q_ij_*}, where *q_ij_* is the instantaneous rate of change from codon *i* to codon *j*.
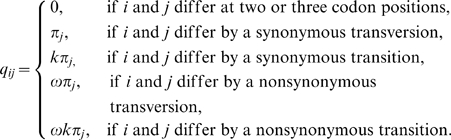
(1)Here, *k* is the transition/transversion rate, 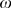 is the nonsynonymous/synonymous rate ratio (*dN*/*dS*), and *π*_j_ is the equilibrium frequency of codon *j*, which can be estimated from the sequence data by several models [Fequal, F1×4, F3×4, and F61, reviewed in 38]. Parameters *π*_j_ and *k* characterize the pattern of mutations, whereas 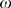 characterizes selection on nonsynonymous mutations. *Q_codon_* is used to calculate the transition-probability matrix
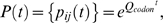
(2)where *p_ij_*(*t*) is a probability that a given codon *i* will become *j* after time *t*. Parameters *k*, *t*, and 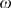 are estimated by maximization of the log-likelihood function
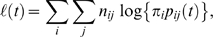
(3)where *n_ij_* is the number of sites in the alignment consist of codons *i* and *j*. The estimated parameters are then used to calculate *dN* and *dS* [38].

### A new method for the simultaneous estimation of selection intensities in overlapping genes

We follow the maximum likelihood approach of Goldman and Yang [37], [38] to construct a model that accounts for different selection pressures on the genes in the overlap. We start with the simplest case, that of opposite-strand phase-0 overlaps. The reason this is the simplest case is that each codon overlaps only one codon in the overlapping gene. The substitution of nucleotides in opposite-strand phase-0 overlaps is specified by the substitution-rate matrix, *Q_codon_* = {*q_ij_*}, where *q_ij_* is the instantaneous rate of change from codon *i* to codon *j*.
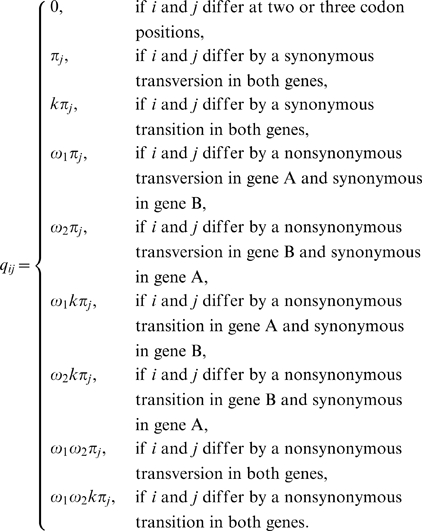
(5)


The main difference between this model and the single-gene model is that here we distinguish between two *dN*/*dS* ratios (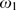 and 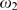 for gene 1 and gene 2, respectively). Another difference is the estimation of codon-equilibrium frequencies. Since the parameters of codon frequencies characterize processes that are independent of the selection on overlapping regions, we estimate these frequencies using the non-overlapping regions of each gene. The calculation of the transition-probability matrix and the log-likelihood function is done in the same way as in the single-gene model (equations 2 and 3).

**Figure 2:**
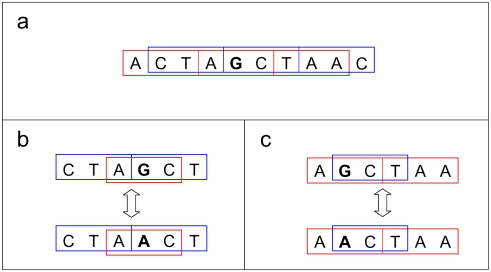
a. An overlapping gene pair (red and blue). b. The codon that is allowed to evolve is marked in red. The substitution in the second-codon position affects the overlapping codon in blue. c. The opposite situation in which only the codon marked in blue is allowed to change.

The above model is a simple expansion of the single-gene model to account for opposite-strand overlaps in phase 0. However, this model cannot be used in the other four overlap cases, same-strand phase-1 and phase-2 overlaps and opposite-strand phase-1 and phase-2 overlaps, because in all these cases a codon overlaps two codons of the second gene. Therefore, we set the unit of evolution to be a codon (the reference codon) and its two overlapping codons, which together constitute a sextet (Figure 2). The sextet is, therefore, the smallest unit of evolution in overlapping genes. In our model, each gene constitutes a set of sextets and within each sextet, only the reference codon is allowed to evolve. Changes in this codon affect the two overlapping codons. For example, consider the red and blue overlapping genes in Figure 2a. A change from G to A in position five (Figure 2a, bold) is illustrated in Figure 2b for the red gene as a reference and in Figure 2c for the blue gene as a reference. Restricting changes to the reference codon only is essential for the model, since changes outside the reference codon will require the consideration of other overlapping codons outside of the sextet, and so *ad infinitum*. In addition, this restriction allows the model to maintain the assumption that each reference codon evolves independently. For gene A as the reference gene, we specify the substitution-rate matrix, *Q^A^_sextet_* = {*q^A^_uv_*} where *q^A^_uv_* is the instantaneous rate from sextet *u* to sextet *v* with the codons of gene A as the reference codons:
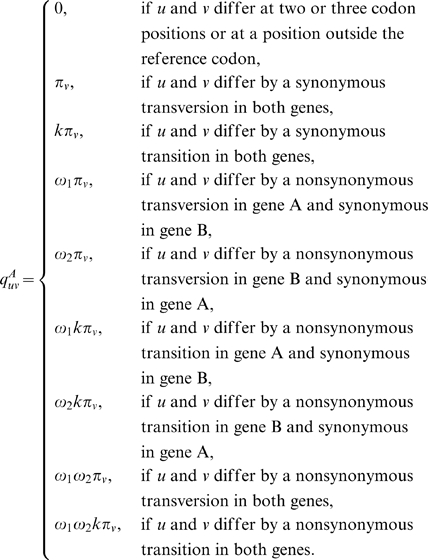
(6)Similarly, we specify the substitution-rate matrix, *Q^B^_sextet_* = {*q^B^_uv_*} for gene B as the reference gene, where *q^B^_uv_* is the instantaneous rate from sextet *u* to sextet *v* with gene B codons as the reference codons. These substitution-rate matrixes, *Q^A^_sextet_* and *Q^B^_sextet_*, can be used to calculate transition-probability matrixes (equation 2). However, these transition-probability matrixes cannot be used directly in the maximization of a log-likelihood function (equation 3) because they do not allow changes between any two sextets (as required in a Markov process). For example, the transition probability between sextets AAAAAA and CAAAAA (where the reference codons at positions 3-5 are underlined) would be zero for any given time *t*, because changes at a position outside of the reference codon are not allowed. A similar difficulty led Pedersen and Jensen [36] to use a complicated, computationally-expensive, simulation procedure to estimate model parameters. Hence, we use *Q^A^_sextet_* and *Q^B^_sextet_* to construct codon-based substitution-rate matrixes 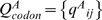 and 
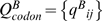
 by summing the rates over all sextets that share the same reference codon. Similar approach was used by Yang et al. [39] to construct an amino acid substitution-rate matrix from a codon substitution-rate matrix. Let *I* and *J* represent the sets of sextets whose reference codons are *i* and *j*, respectively, than, the substitution rate from codon *i* to codon *j* is
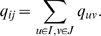
(7)
*Q^A^_codon_* and *Q^B^_codon_* are used to calculate a transition-probability matrix for each of the genes as in equation 2.
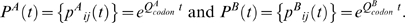
(8)


The new transition-probability matrixes are suitable for a maximization of a log-likelihood function since they allow transition between each two codons. *P^A^*(*t*) and *P^B^*(*t*) can be used separately to estimate model parameters in a log-likelihood function for each gene (equation 3). However, in order to use all the information in the data, we combine the two transition-probability matrixes to create the following log-likelihood function:
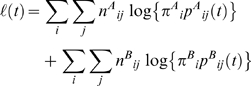
(9)


Here, *π* ^A^_i_ and *π* ^B^_i_ are the equilibrium frequency of codons in gene A and gene B respectively, estimated from the non-overlapping regions of the genes. *n^A^_ij_* and *n^B^_ij_* are the number of sites in the alignment consist of codons *i* and *j* for gene A and gene B, respectively.

The method was implemented in Matlab and is available at http://nsmn1.uh.edu/~dgraur/Software.html. Running time is ∼7 seconds for a pair of aligned sequences of length 1000 codons. Similar to the single-gene model, this method can be extended to deal with multiple sequences in a phylogenetic context and to test hypotheses concerning variable selection pressures among lineages and sites [40]–[42].

## Results

### Simulation studies

We tested the performance of our new method for simultaneous estimation of selection intensities in comparison to the independent estimation that does not account for gene overlap (as described in equation 1). We examined the effects of nonsynonymous/synonymous rate ratio in each gene (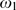 and 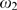), transition/transversion rate ratio (*k*), and sequence divergence (*t*). In all of the methods, we used the F3×4 model [38] to estimate codon equilibrium frequencies. For each set of parameters, we generated 100 replications of random overlapping gene pairs (each gene was 2000 codons in length with 1000 codons in the overlap) by sampling codons from a uniform distribution of sense codons. To simulate the evolution along a branch of length *t*, we divided the sequence of the overlapping gene pair into three regions: non-overlapping region of gene one, non-overlapping region of gene two, and overlapping region. For the non-overlapping regions, we calculated the transition-probability matrixes based on the non-overlapping model in equation 1. For the overlapping region, we calculated the transition-probability matrixes (based on the overlapping models in equations 5 and 6). Using the three probability matrixes, we simulated nucleotide substitutions at each codon independently [38].

### Different selection pressures

**Figure 3:**
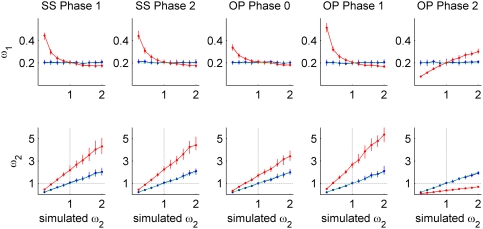
Simulation results in same-strand (SS) and opposite-strand (OP) overlaps. Estimations of the ratios of nonsynonymous to synonymous rates in the two genes (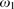 and 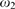) by simultaneous estimation (blue line) and by independent estimation (red line) are plotted against the true value (X axis, dashed green line) for five types of overlap. The simulated value of 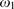 was set to 0.2 and 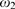 was varied between 0.2 and 2. *k* was set to 1 and *t* was set to 0.35. Each data point is the median of 100 replications. Vertical lines mark the lower and upper quartiles. Top: estimation of 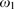. Bottom: estimation of 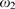. Dotted black lines (X = 1 and Y = 1) illustrate the range of parameters that result in false inference of positive selection by independent estimation, i.e., when simulated 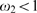 and estimated 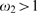.

To examine the effect of different selection pressures, we initially set *k* = 1 and *t* = 0.35, which result in a sequence divergence of ∼10%. We set 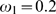 and varied 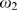 between 0.2 and 2. In Figure 3, we compare the simultaneous estimation of 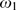 and 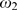 (blue line) and the independent estimation (red line) to the true simulated value (X axis, dashed green line) in the five types of overlaps. Each data point is the median of 100 replications. We use the median rather than mean since ratios are not normally distributed. In all overlap types, the estimation of our method is in near-perfect match to the simulated value (blue and green lines, Figure 3) and the bias in the independent estimation of 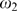 is greater than that of 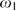.

As expected, we found a similar pattern of bias in all overlap types except opposite-strand phase 2. In all of these overlap types (same-strand phase 1, same-strand phase 2, opposite-strand phase 0, and opposite-strand phase 1), the independent estimation of 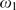 is overestimated for 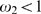 and underestimated for 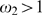. The independent estimation of 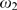 is overestimated throughout the range of the simulation resulting in the false inference of positive selection in gene 2, while in reality this gene is under weak purifying selection. For example, the independent estimation of 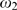 in same-strand phase 1 is greater than one (apparent positive selection) for simulated values of 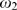 between 0.5 and one.

The bias in opposite-strand phase 2 differs from the other overlap types because this overlap contains positions that are synonymous in both genes (Figure 1). Because of this factor, the independent estimation of 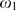 is underestimated for 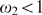 and overestimated for 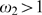. The independent estimation of 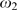 is underestimated throughout the range of the simulation, resulting in inability to detect positive selection in gene 2 for simulated values of 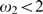.

To compare the magnitude of error in the independent estimation of each overlap type, we set *k* = 1, *t* = 0.35, 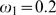, and 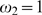. We calculated the mean square error (MSE) for the independent estimation of 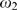 (the parameter whose estimation is most biased) in each overlap type. We use MSE because it measures both the bias and the variance. The most biased type is opposite-strand phase 1 followed by both same-strand phase 1 and phase 2, opposite-strand phase 0, and opposite-strand phase 2 (Table 1). As expected, the magnitude of error among overlap types is correlated with the proportion of sites in each overlap type that are synonymous in one gene and nonsynonymous in the overlapping genes (Table 1).

**Table 1:** The mean square error (MSE) of the independent estimation of selection intensity is correlated with the proportion of changes that are synonymous in one gene and nonsynonymous in the overlapping gene (SN changes).

Orientation	Phase	Proportion of SN changes	MSE Independent	MSE Simultaneous
Same-Strand	1	47%	1.83	0.04
	2	47%	1.94	0.05
Opposite-Strand	0	43%	0.64	0.03
	1	63%	3.23	0.06
	2	39%	0.40	0.04

### Transition/transversion rate ratio and sequence divergence

**Figure 4:**
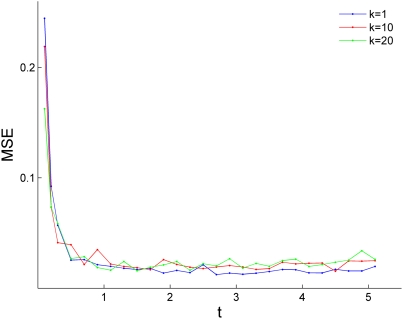
The influence of transition/transversion rate ratio (*k*), and sequence divergence (*t*) on the performance of the new method. The mean square error (MSE) is plotted against *t* for *k* = 1, 10, and 20 (blue, red, and green, respectively).

We tested the influence of transition/transversion rate ratio (*k*), and sequence divergence (*t*) on the performance of the new method for simultaneous estimation. Focusing on same-strand phase 1, we set 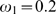, 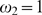 and vary *k* between 1 and 20, and *t* between 0.1 and 1.1. We calculated the MSE for the estimation of 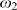. The results of 100 replications suggest that transition/transversion rate ratio does not affect the accuracy of the method, whereas the accuracy of the method is reduced for *t*≤0.3 (sequence divergence of ∼8% or less, Figure 4). We note that although our method performs well in high sequence divergence, the inference of selection can be biased by the reduced quality in alignment of distant sequences.

### Testing the new estimation method on genes from influenza H5N1 and H9N2 strains

We used the new method to estimate selection pressures in two cases of overlapping genes in avian influenza A. We chose PB1-F2 and NS1 genes (which overlap with PB1 and NS2, respectively), because they were previously reported to exhibit values of dN/dS indicative of positive selection [19], [20], [25], [26]. For each gene, we collected all the annotated gene sequences from the two most sequenced subtypes, H5N1 and H9N2 from the NCBI Influenza Virus Resource [43]. Within each subtype set, we aligned the overlapping regions of all gene pairs at the amino acid level using the Needleman-Wunsch algorithm [44]. We used all pairwise alignments with sequence divergence greater than 5% (since estimation is less accurate at low divergence rates) to estimate selection intensities either simultaneously or independently (Table 2). Using higher cutoffs for sequence divergence did not affect the results (data not shown). Pairs in which the independent estimation of dS was zero (leading to infinity value for dN/dS) were excluded. In agreement with previous studies, PB1-F2 and NS1 genes appear to be under positive selection when gene overlap is not accounted for. However, by using our new method for simultaneous estimation, these genes seem to be under weak purifying selection. As predicted by our simulation, the bias in the independent estimation is dependent on the degree of purifying selection acting on the overlapping gene, leading to higher bias in PB1-F2 compared to NS1.

**Table 2:** Estimation of selection intensity (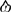) by independent and simultaneous estimation.

Gene	Subtype a	Independent 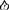 b ^, ^ c	Simultaneous 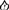 b
NS1	H5N1	**1.25** (0.75 1.93)	0.81 (0.41 1.52)
	H9N2	**1.46** (1.07 2.24)	0.58 (0.38 0.86)
NS2	H5N1	0.34 (0.24 0.52)	0.32 (0.22 0.50)
	H9N2	0.24 (0.15 0.35)	0.23 (0.13 0.36)
PB1-F2	H5N1	**6.75** (5.74 9.88)	0.52 (0.40 0.76)
	H9N2	**6.41** (5.52 7.92)	0.46 (0.34 0.75)
PB1	H5N1	0.03 (0.02 0.05)	0.02 (0.02 0.04)
	H9N2	0.03 (0.02 0.05)	0.02 (0.01 0.04)

a Number of pairwise alignments of NS1 – NS2 overlaps is 10,569 and 8,745 for H5N1 and H9N2 subtypes, respectively; Number of pairwise alignments of PB1-F2 – PB1 overlaps is 16,112 and 33,720 for H5N1 and H9N2 subtypes, respectively.

b Median of 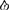 over all pairwise comparisons. Lower and upper quartiles are noted in parentheses.

c Values of selection intensity in PB1-F2 and NS1 genes that appear as positive selection by independent estimation are bolded.

## Discussion

Overlapping genes are widespread in all taxa, but are particularly common in viruses [45]. The sequence interdependence imposed by gene overlap adds complexity to almost any molecular evolutionary analysis. Here, we presented a new method for the estimation of selection intensities in overlapping genes. By simulation, we verified the accuracy of the method, tested its limitations, and compared the possible outcomes of estimating selection without accounting for gene overlap across different overlap types. We find that estimating selection as if the genes are independent of one another results in the false appearance of positive selection. Our model can be used to identify true functional genes, which are usually under negative or positive selection, from among hypothetical overlapping ORFs, which are mainly spurious.
